# Infants’ dietary arsenic exposure during transition to solid food

**DOI:** 10.1038/s41598-018-25372-1

**Published:** 2018-05-08

**Authors:** Antonio J. Signes-Pastor, Kathryn L. Cottingham, Manus Carey, Vicki Sayarath, Thomas Palys, Andrew A. Meharg, Carol L. Folt, Margaret R. Karagas

**Affiliations:** 1 Department of Epidemiology, Geisel School of Medicine, Dartmouth College, 1 Medical Center Dr., 7927 Rubin Bldg., Lebanon, NH 03756 USA; 2 Department of Biological Sciences, Dartmouth College, Hanover, NH 03755 USA; 3 Institute for Global Food Security, Queen’s University Belfast, David Keir Building, Malone Road, Belfast, BT9 5BN Northern Ireland UK; 4 University of North Carolina, Chapel Hill, North Carolina, USA; 5 Children’s Environmental Health and Disease Prevention Research Center at Dartmouth, Lebanon, USA

## Abstract

Early-life exposure to inorganic arsenic (i-As) may cause long-lasting health effects, but as yet, little is known about exposure among weaning infants. We assessed exposure before and during weaning and investigated the association between solid food intake and infants’ urinary arsenic species concentrations. Following the recording of a comprehensive 3 day food diary, paired urine samples (pre- and post-weaning) were collected and analyzed for arsenic speciation from 15 infants participating in the New Hampshire Birth Cohort Study. Infants had higher urinary i-As (*p*-value = 0.04), monomethylarsonic acid (MMA) (*p*-value = 0.002), dimethylarsinic acid (DMA) (*p*-value = 0.01), and sum of arsenic species (i-As + MMA + DMA, *p*-value = 0.01) during weaning than while exclusively fed on a liquid diet (i.e., breast milk, formula, or a mixture of both). Among weaning infants, increased sum of urinary arsenic species was pairwise-associated with intake of rice cereal (Spearman’s ρ = 0.90, *p*-value = 0.03), fruit (ρ = 0.70, *p*-value = 0.03), and vegetables (ρ = 0.86, *p*-value = 0.01). Our observed increases in urinary arsenic concentrations likely indicate increased exposure to i-As during the transition to solid foods, suggests the need to minimize exposure during this critical period of development.

## Introduction

Arsenic is a ubiquitous element in the environment and occurs in different oxidation states with measurable levels in the food chain of i-As (inorganic arsenic), including arsenite and arsenate, as well as various organoarsenicals^1–5^. Biomethylation of i-As to monomethylarsonic acid (MMA) and dimethylarsinic acid (DMA) before excretion in urine is considered the main metabolic mechanism of i-As detoxification^6–8^; as such, urinary arsenic species concentrations are considered a reliable biomarker of i-As exposure^9^. However, urinary DMA and MMA from other sources besides i-As biomethylation (i.e. direct exposure from diet or due to biotransformation of more complex organosenicals) may lead to overestimation of i-As from measures of total urinary arsenic or the sum of the urinary species^10,11^. Intake of i-As is an established cause of cancers of the lung, skin, and bladder and a possible cause of others, with accumulating evidence of effects on non-cancer health outcomes such as neurological, cardiovascular, respiratory and metabolic diseases^12,13^. A growing body of evidence points to the heightened vulnerability of infants to lifelong health impacts of i-As, and, thus early life i-As dietary exposure is an increasingly recognized public health concern even for populations with access to drinking water with low arsenic concentrations^1,14–18^. Health effects associated with direct exposure to organic forms of arsenic - including DMA and MMA - are less certain, and arsenobetaine (AsB), which predominates in fish and seafood products, is excreted in the urine unchanged, and, therefore considered non-toxic^4,5,10,19^.

Prior to weaning, infants experience very little exposure to i-As from breastfeeding. Even in areas with drinking water containing very high concentrations of arsenic, only small amounts appear to pass through mammary glands to breast milk^4,20,21^. In prior work, we found a median total arsenic concentration of 0.31 μg/L in breast milk from mothers in the New Hampshire Birth Cohort Study^22^, with about 10% of households having tap water exceeding the U.S. EPA and WHO maximum contaminant level of 10 μg/L of i-As^23,24^. Formula powder contains predominantly i-As with a total arsenic level of up to 12.6 μg/kg^25^, which would lead to an arsenic content in ready-to-eat formula of about 20-fold higher than the median content in breast milk reported earlier^22^ if arsenic-free water is used for formula powder reconstitution with a formula powder:water ratio 1:1. Higher i-As exposures occur when arsenic-contaminated drinking water is used. Thus, formula fed-infants tend to have a higher level of urinary arsenic species associated with i-As exposure than exclusively breastfed infants. Still, the overall urinary arsenic concentrations and estimated i-As exposure have been reported to be relatively low compared to the general population^15,20,22,26^.

Little is known about infant’s dietary i-As exposure during weaning to solid foods. In a study of 11 infants from Belfast, Northern Ireland, concentrations of urinary i-As, MMA, and DMA were each higher during weaning to solid foods^15^. While specific dietary components were not evaluated, the vast majority of infants were reported to have consumed rice, a known source of i-As exposure due to the enhanced arsenic mobility in paddy-managed soils^15,27^. Appreciable concentrations of i-As and DMA up to 323 and 297 μg/kg, respectively - and traces of MMA have been found in rice products commonly eaten during weaning such as baby rice, rice cereals and rice crackers^16,28,29^. Concentrations of i-As of up to 20 μg/kg have also been found in popular fruit and vegetable purees, and to 49 μg/kg in mixed cereals containing wheat, barley, oat, rye, sorghum, and/or millet marketed for weaning infants^25,29^. As reviewed previously, concentrations of i-As averaging 5 to 20 μg/kg have been detected in infant foods that contain apples, including apple juice, mixed juices and vegetables^30^.

Given the potential for i-As exposure during this critical period of development, we sought to determine concentrations of urinary arsenic species during the weaning period, and their relation to intake of specific foods in a U.S. pregnancy cohort study. We hypothesized that urinary arsenic species concentrations associated with i-As exposure would increase during weaning due to consumption of foods containing i-As such as rice, mixed cereals, apples, and vegetables reflecting increased exposure to this toxic form of arsenic.

## Results

### Study population

The study population comprised 8 girls and 7 boys. Infants’ mothers did not smoke during pregnancy, and only one participant reported secondhand smoke during pregnancy. Selected characteristics of the study population are depicted in Table 1.

**Table 1:** Selected characteristics of the study population.

Selected characteristics	*n* [%] or *n* [median (min–max)]	
**Infants’ sex:**		
Female	8 [53%]	
Male	7 [47%]	
Maternal relationship status, married	15 [100%]	
Maternal age at enrolment (years)	15 [31 (25–39)]	
Pre-pregnancy BMI (kg/m^2^)	15 [24.9 (20.8–35.5)]	
Gestation age (weeks)	15 [40.1 (37.6–41.7)]	
**Number of previous live births:**		
0	7 [47%]	
>1	8 [53%]	
No maternal smoke during pregnancy	15 [100%]	
**Maternal secondhand smoke during pregnancy**		
No	14 [93%]	
Yes	1 [7%]	
**Maternal highest attained level of education:**		
Any post-graduate schooling	7 [47%]	
College graduate	5 [33%]	
Junior college graduate, or some college or technical school	3 [20%]	
**Daily number of infant’s feedings** ^**1**^ **:**	**4 months of age**	**6 months of age**
Breast milk	14 [5.1 (3.3–11.7)]	12 [5.5 (1.0–9.3)]
Formula	4 [2.3 (0.6–8.0)]	5 [3.7 (0.3–7.3)]
Rice cereal		8 [0.3 (0.3–1.3)]
Other cereals		6 [0.7 (0.3–3.3)]
Fruits		11 [0.6 (0.3–3.3)]
Vegetables		8 [0.8 (0.3–1.7)]
Yogurt		2 [0.3]
Other foods^2^		1 [0.3]

^1^Average daily feedings based on a 3 day food diary.

^2^Refers to a participant that reported a meal of turkey with rice without specifying the amount consumed of each product individually.

### Analytical quality control

The NIST Natural Water Standard Reference Material 1640a total arsenic average percentage recovery±SE based on *n* = 5 was 100 ± 0.58%. The urine lyophilized material ClinChek® - Control level I average percentage recovery ± SE based on *n* = 6 was 108 ± 0.03%, 99 ± 0.01%, 98 ± 0.03%, and 91 ± 0.05% for i-As, MMA, DMA, and AsB, respectively. The average coefficient of variation for six masked replicate urine samples was 5.8% for i-As, 2.4% for MMA, 5.6% for DMA, and 4.5% for AsB. The limit of detection (LOD) for total arsenic in water and arsenic speciation analyses in urine was 0.011 μg/L.

The infants’ urine samples had a low median specific gravity value (range) of 1.004 (1.002–1.011) g/mL, and 1.006 (1.003–1.014) g/mL at 4 and 6 months of age, respectively. Thus, a nearly perfect correlation was observed between urinary arsenic species concentrations with and without specific gravity adjustment (Spearman’s ρ = 1, *p*-value < 0.001). This finding suggested that adjustment for urinary dilution is not necessary, as indicated in both our sensitivity analysis (data not shown) and previous studies^31–33^.

### Food diary

At 4 months of age, all study infants were exclusively fed with a liquid diet including breast milk, formula, or a mix of the two; no food diaries reported solid food intake. For the four infants consuming formula, water was used for formula powder reconstitution (three used home tap water, and one used bottled water) with a daily median intake of 172.5 mL (Table S1). Of these four, one infant was exclusively formula fed and the three others consumed a mixture of breast milk and formula. The overall median daily number of breast feedings was 5.1 (*n* = 14), and the median daily formula powder intake was 92.4 g based on the four formula-fed or mixed-fed infants (Tables 1 and S1).

At the age of 6 months, all 15 infants reported some solid food consumption as well as breast milk (*n* = 10), formula (*n* = 3), or a mixture of both breast milk and formula (*n* = 2). Eight infants were reported to consume water either through prepared solid foods or reconstituted formula powder (*n* = 7 from the home tap and *n* = 1 bottled water) with a median daily intake of 269.3 mL (Table S1). The median number of daily breast feedings was 5.5 (*n* = 12) (Table 1). The median daily amount of formula powder, rice cereals, other cereals, fruits, and vegetables consumed was 101.3 g (*n* = 5), 18.5 g (*n* = 5), 40.2 g (*n* = 6), 33.0 g (*n* = 9), and 42.5 g (*n* = 7), respectively (Table S1). Intake of rice cereal was unrelated to intakes of any of the other food categories (ρ = 0.21, *p*-value = 0.52, ρ = 0.06, *p*-value = 0.87, and ρ = 0.41, *p*-value = 0.21 for other cereals, fruits, and vegetables, respectively) or with total solid food intake (ρ = 0.28, *p*-value = 0.40). Fruit and vegetable consumption were only weakly associated (ρ = 0.49, *p*-value = 0.09). Intakes of formula powder, fruits, vegetables, and other cereal were each positively correlated with intake of total solid food (ρ = 0.75, *p*-value = 0.003; ρ = 0.76, *p*-value = 0.004; ρ = 0.84, *p*-value < 0.001; and ρ = 0.85, *p*-value < 0.001, respectively). In contrast, number of breast feedings was inversely correlated with the amount of total solid food consumption (ρ = −0.71, *p*-value = 0.006) (Figure S1).

Solid food intake (in grams) largely comprised fruits and vegetables, with an average contribution percentage of 49% and 23%, respectively, followed by rice cereals (20%) and other cereals (12%) (Table S1).

### Urinary arsenic and dietary exposure assessment

At 4 months of age, concentrations of all individual arsenic species were higher than the LOD except for the urinary MMA; 13 of the 15 infants had MMA concentrations higher than the LOD. Infants who were exclusively breastfed at 4 months of age had lower median urinary arsenic concentrations compared to those who were partially or exclusively fed with formula (1.6, 11.4, 6.2, and 4.9-fold lower median urinary concentrations of i-As, MMA, DMA and sum of urinary arsenic species excluding AsB (i-As + MMA + DMA), respectively) (Table S2). A median AsB concentration of 0.061 μg/L was found in infants’ urine at the age of 4 months (Table S2).

**Figure 1:**
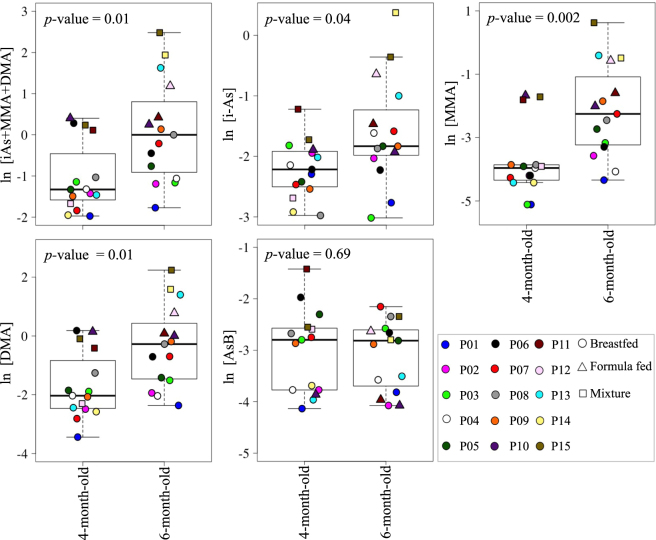
Concentrations of urinary arsenic species before (at 4 months of age) and during weaning (at 6 months of age) to solid foods. At 6 months of age infants’ diets included solid food in addition to breast milk, formula, or a mixture of both breast milk and formula. Each infant is identified by a unique color to facilitate the comparison between the two time points. The *p*-values were derived from paired Wilcoxon signed-rank test analyses. Urinary arsenic species concentrations were natural logarithm transformed to help visualization.

At 6 months of age, during weaning, all individual species of arsenic were higher than the LOD. Weaning infants had a 1.5, 5.5, 5.8, and a 3.8-fold higher median urinary i-As, MMA, DMA and sum of urinary arsenic species, respectively, compared to infants at 4 months of age. The paired Wilcoxon signed-rank test *p*-values were statistically significant for i-As (*p*-value = 0.04), MMA (*p*-value = 0.002), DMA (*p*-value = 0.01) and sum of urinary arsenic species (*p*-value = 0.01). Concentrations of urinary AsB did not differ between the two ages (*p*-value = 0.69) (Fig. 1 and Table S2).

**Figure 2:**
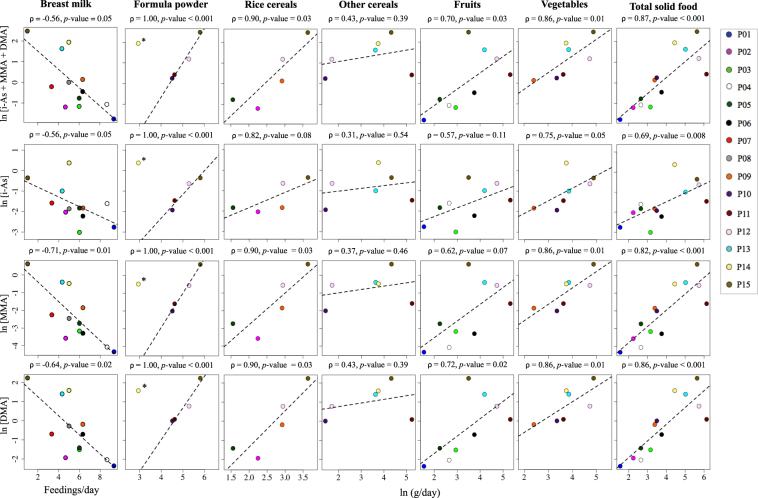
Concentrations of infant urinary arsenic species in relation to intake of specific foods at 6 months of age. Each infant is identified by a unique color. Spearman’s correlation coefficient and *p*-value for each dietary item. Urinary arsenic species concentration and solid food consumption were natural logarithm transformed to help visualization. The least-squares regression line is also included to help visualize associations. *Infant P14 was not included in Spearman’s correlation between urinary arsenic species content and the formula powder intake level (outlier).

Estimated arsenic exposure through water consumption was low, with a median of 0.002 μg/day and 0.010 μg/day at the infants’ age of 4 (*n* = 4) and 6 months (*n* = 8), respectively. This is consistent with the low arsenic concentrations in both household tap water (median of 0.102 μg/L, *n* = 15) for this cohort and in bottled water (reported values of <1.4 μg/L)^34^. One infant (P14) had a 80-fold higher water arsenic concentration (8.176 μg/L) compared to the median concentration for all infants (0.102 μg/L) and, therefore, was considered an outlier in the analysis of formula powder intake in relation to infants’ urinary arsenic concentrations (Fig. 2 and Table S3). After excluding infant P14, formula powder intake was positively correlated with individual urinary arsenic species concentrations and the sum of urinary arsenic species at the age of 6 months (*p*-value < 0.001) (Fig. 2).

Of the weaning foods, rice cereal, fruit, and vegetable consumption were each positively correlated with urinary sum of arsenic species (ρ = 0.90, *p*-value = 0.03; ρ = 0.70, *p*-value = 0.03; and ρ = 0.86, *p*-value = 0.01, respectively). Similar trends were observed for each of the individual species and were statistically significant for DMA (ρ = 0.90, *p*-value = 0.03; ρ = 0.72, *p*-value = 0.02; and ρ = 0.86, *p*-value = 0.01, respectively) and MMA for rice cereal and vegetable consumption (ρ = 0.90, *p*-value = 0.03 and ρ = 0.86, *p*-value = 0.01, respectively). At 6 months of age, the number of daily breast feedings was inversely associated with urinary MMA and DMA (ρ = −0.71, *p*-value = 0.01 and ρ = −0.64, *p*-value = 0.02, respectively) and to a lesser extent with urinary i-As and the sum of urinary arsenic species (ρ = −0.56, *p*-value = 0.05 for both) (Fig. 2). The inverse trend between the number of daily breast feedings and urinary arsenic species during weaning held even after adjusting for solid food intake (ρ = −0.64, *p*-value = 0.04 for the sum of urinary arsenic species, and ρ = −0.60, *p*-value = 0.07 for urinary i-As).

## Discussion

In our study population, with relatively low i-As exposure through water consumption, infants’ transition from a liquid diet to solid food was associated with increased urinary arsenic concentrations of i-As, MMA and DMA. Higher intake of rice cereals, fruits, and vegetables were each related to increased concentrations of urinary sum of arsenic including i-As, MMA and DMA during weaning. Our findings further suggest that infants’ dietary exposure to i-As before weaning, at the age of 4 months, was lower in exclusively breastfed infants compared to that for formula-fed, which agrees with previous studies including those using low-arsenic water for formula powder reconstitution^15,22,35^.

At about 6 months of age, infants gradually transition to solid food to meet energy and nutrient demands. We hypothesized that this would increase the exposure to toxic elements such as i-As^35,36^. Indeed, we found a 3.8-fold increase in the median urinary sum of arsenic species concentrations in infants during early weaning. Our findings agree with those in the prior study of 11 infants from Belfast, Northern Ireland, where a 4.9-fold higher median urinary sum of arsenic species concentration was found in paired samples from weaning infants at the median age of 7.7 months compared to that before weaning at the median age of 2.1 months^15^. The majority of infants in the Belfast study were reported to have eaten rice cereals, although the precise nature of their diets was not ascertained.

Fish and seafood consumption is considered the main source of AsB, a putative non-toxic form of arsenic excreted unchanged in urine rapidly, with the largest share excreted within a few hours after ingestion^5,10,19,37^. Despite the fact that infants consumed neither fish nor seafood in the 3 days prior to the urine sample collection, traces of AsB were observed in their urine with no changes before and after weaning, which suggests that breast milk and formula may contribute to that exposure. Indeed, a recent study has reported that AsB is among the main arsenic species in breast milk^38^.

Our current study included a detailed dietary diary, and as expected, suggests that urinary concentrations of arsenic species associated with i-As exposure increase with rice consumption during the weaning period. Concerns regarding i-As content in rice destined for the production of food for infants and young children have already led to an “action level” of 100 μg/kg of i-As in infant rice cereals proposed by the U.S. FDA^39^, paralleling the maximum level established by law in the E.U.^40^. Rice also may contain varying concentrations of DMA and MMA depending on the geographical origin of the rice. Thus rice itself may directly contribute to DMA and MMA exposure^41,42^ and the sum of the urinary arsenic species may not exclusively reflect dietary i-As exposure. Our findings of a positive trend of rice cereal intake with the sum of urinary arsenic species and the individual fractions, including i-As are in agreement with prior studies including our own study of 12 month old children, and a study with children of 5 to 8 years old from Uruguay^16,43^. However, to our knowledge this is among the only studies of urinary arsenic species concentrations in longitudinally paired infant samples both pre- and post-weaning to rice cereal intake.

Arsenic in fruits and vegetables also is of concern. Detection of i-As in apple juice, from past residual arsenical pesticide use, led the U.S. FDA to propose an “action level” of 10 μg/L, which coincides with the drinking water Maximum i-As Contaminant Level set by the U.S. EPA^24,44^. The maximum i-As level of 100 μg/kg has been broadly set for canned or processed complementary food for infants and young children in China^45^. Additional regulations to tightly limit the i-As content in other specific foodstuffs such as fruits or vegetables including those specifically marketed to infants do not yet exist despite ongoing occurrence data on i-As in various food commodities in the E.U.^46^ and U.S.^4,25,47^, and our findings here that suggest these foods may increase biomarker concentrations of i-As among infants. In our study, fruit and vegetable consumption together comprised a large portion of infants’ total solid food intake. The amount of fruits and vegetables consumed by each infant were uncorrelated. A strong association was found between urinary sum of arsenic species content and consumption of both fruits and vegetables. While low i-As concentrations have been reported in fruits and vegetables targeted for infants^25,30^, infants also consume other fruits and vegetables that may contain appreciable i-As concentrations if grown in a contaminated agricultural environment^14,48–50^.

There are limitations to our study that need to be recognized. First, our study was based on a small subsample of the New Hampshire Birth Cohort Study, and, therefore, may have lacked statistical power for foods that were not consumed by many infants. Moreover, the urine sample collection marked the end of recording dietary information on the third day of the 3 day food diary; however, depending on when it took place the reported infants’ amount of food and water intake over the 3 day period may be underestimated. Daily urine samples collection would have been ideal; however, that was not possible given what is involved in obtaining an adequate infant urine sample. Additionally, direct dietary exposure to DMA and MMA was not assessed in this study, and thus as mentioned weaning infants’ increased urinary sum of arsenic species compared to their urinary sum of arsenic species when consuming only liquids may not exclusively reflect exposure to dietary i-As. Although our findings are based on a limited sample size, we observed a statistically significant increase of infants’ urinary sum of arsenic species during transition to solid food as well as specific species.

In conclusion, our findings highlight the importance of ensuring low i-As containing weaning foods in addition to research into the adverse health risks related to early-life dietary i-As exposure, particularly during this critical period of development. These results also may help to provide a framework for understanding early-life dietary exposure to arsenic, including i-As, and strategies to protect infants from potential adverse health effects later in life^1,14–18^.

## Methods

### Study population

Our study population comprised 15 infants followed as part of the New Hampshire Birth Cohort Study (NHBCS) – a mother-child prospective cohort study. Only infants with an exclusively liquid diet (breast milk, formula, or mixture of both breast milk and formula) at the age of 4 months, whose transition to solid food started at the age of 6 months, and with paired urine samples were included in the study. The general design of the NHBCS has been described previously^18,51^. Briefly, pregnant women from 18 to 45 years of age receiving prenatal care at the study clinics in the state of New Hampshire and whose households were served by a private, unregulated water system, not planning to change residency before delivery and carrying a singleton pregnancy were recruited beginning in January 2009. Women were at approximately 24 to 28 weeks of gestation at enrollment at which time a medical history and a lifestyle questionnaire was administered to gather sociodemographic, reproductive, and health history information. Our Institutional Review Board (IRB) was the Committee for the Protection of Human Subjects at Dartmouth College, and they approved the protocol of the study. All methods were performed in accordance with the relevant guidelines and regulations, and all participants provided written informed consent in accordance with guidelines from the Committee.

### Food diary

At the age of 4 and 6 months, infants’ parents or caregivers were asked to complete a 3 day food diary prior to urine collection. A 3 day food diary form was provided to infants’ parents or caregivers along with detailed instructions for self-completion with examples that described how to properly complete the diary entries including details about measuring units. The 3 day food diary template and instructions are included in the supplementary information. The infant food diary included every food or beverage consumed during three consecutive days. Each day recorded on the food diary started with the first meal after midnight and ended just before midnight at 11:59 p.m. The food diary included the type of food consumed, a brief description of how the food was prepared and the amount ingested. For all formulas and ready-made baby foods, the brand and exact commercial product name were recorded, which allowed us to gather further information about their labeled ingredients after registration. The amounts of each food item was reported in the diary in grams, ounces, cups, tablespoons, and scoops, and then were converted to mL (water) or grams (formula powder and solid food items) prior to analysis. We considered 1-ounce equal to 29.57 mL for water and 28.35 g for formula powder and solid foods; 1-ounce was estimated to equal 1/8 cups, 2 tablespoon and 6 teaspoons. Further, according to manufacturer directions 1-scoop of formula powder equaled 8.3 g. The 3 day food diary also asked whether water was used and if so, the source of the water, i.e., household tap water, bottled water or other sources. The dietary information recorded on the 3 day food diary was classified into the following categories for analysis after registration: (i) water, including that added to formula powder and to prepared solid foods; (ii) breast milk; (iii) formula powder; (iv) rice cereals; (v) other cereals, including oat, wheat, and quinoa; (vi) fruits, including apples, cherries, avocado, mango, watermelon, pears, kiwi, blueberries, peaches, banana, and strawberries; (vii) vegetables, including broccoli, green beans, sweet potato, winter squash, and carrots; (viii) yogurt; (ix) and other foods. Consumption in each category was summed across the 3 day and then divided by 3 to determine the average daily consumption.

### Water and infant urine sample collection and preparation

Household water samples were collected at enrollment. A commercially washed, mineral free, high-density polyethylene bottle complying with the U.S. EPA standards was used for water collection. The bottles were kept in clean, sealed bags, and participants were provided with detailed instructions to minimize contamination. Water samples were stored at −20 °C or lower until analysis^22^. Spot urine samples were collected the third day of the 3 day food diary. Urine samples (free of feces) were taken from infants at the age of 4 and 6 months with supplied diapers and cotton pads with a previously tested arsenic content of 0.021 ± 0.007 μg/L (mean ± SE)^22^. The urine-saturated pads were couriered to Dartmouth Hitchcock Medical Center (DHMC) with frozen ice packs. At Dartmouth, urine was squeezed from the cotton pads, divided into aliquots, specific gravity measured using a handheld refractometer (ATAGO® PAL-10S; Atago U.S., Inc.) and frozen at −80 °C^22^. A 1 mL aliquot from each infant in the study along with six masked replicate urine samples for quality control were shipped on dry ice to the Institute for Global Food Security at Queen’s University Belfast (QUB), Northern Ireland, for arsenic speciation analysis.

### Chemical analysis

Household water samples were analyzed for total arsenic concentration by inductively coupled plasma mass spectrometry (ICP-MS) at the Trace Element Analysis Core at Dartmouth using a Quadruple collision cell 7500c Octopole Reaction System ICP mass spectrometer (Agilent) and He as a collision gas to remove polyatomic interferences. Replicate samples of NIST Natural Water Standard Reference Material 1640a with a total arsenic certified value of 8.075 ± 0.070 μg/L (mean ± SD) and blank samples were included in the analysis as quality control measures as previously described^22^. Urinary arsenic speciation analysis, including i-As (the sum of arsenite and arsenate), MMA, DMA, and AsB, was carried out by ion chromatography (IC-ICP-MS) at the Institute for Global Food Security, QUB using a Thermo Scientific IC5000 ion chromatography system, with a Thermo AS7, 2 × 250 mm column and a Thermo AG7, 2 × 50 mm guard column interfaced with a Thermo ICAP Q ICP-MS in collision cell mode. In addition to the masked sample replicates shipped to QUB, the laboratory included replicate samples of the urine lyophilized material ClinChek^®^ - Control level I with a mean and range certified values of 4.55 (2.73–6.37) μg/L for i-As, 2.50 (1.50–3.50) μg/L for MMA, 9.8 (5.88–13.7) μg/L for DMA, and 16.8 (12.6–21.0) μg/L for AsB, and blank samples as quality control following the methodology previously described^15,52^.

### Statistical analysis

The number of reported breast milk feedings was used as an indirect measure of amount of breast milk consumed^26^. The exposure to arsenic during infants’ transition to solid food was evaluated with paired Wilcoxon signed-rank test statistical analyses to accommodate our relatively low sample size; statistical conclusions are robust to the use of parametric vs. non-parametric tests (data not shown). Infants’ urinary arsenic species concentrations, amount of formula and solid food consumption followed skewed distributions, and thus Spearman’s correlation (ρ) was used to determine the relation between urinary arsenic species and infants’ daily breast feedings and daily intake of formula powder, rice cereal, other cereals, fruit, vegetable, and total solid food. The asymptotic *t* approximation method was used to compute the *p*-values of Spearman’s correlations. We assigned the value of zero to food categories not reported as consumed to determine their percentage contribution to the total amount of solid food intake and to calculate the Spearman’s correlations among them. Analyses were conducted both with and without specific gravity adjustment as sensitivity analyses. To estimate arsenic exposure through water the reported daily-ingested amount was multiplied by its arsenic content (i.e., the measured home tap water arsenic concentration or 1.4 μg/L for bottled water). Half the LOD value was assigned for statistical analyses of the data when samples were below the LOD. All analyses were carried out with the R software for statistical computing^53^.

### Data availability

Analytic data used in this study are included in the manuscript figures and tables and its Supplementary Information files.

## Electronic supplementary material

Supplementary Information
