# Supplementary material for: Infants’ dietary arsenic exposure during transition to solid food
Source: Sci Rep. 2018 May 8;8:7114. doi: 10.1038/s41598-018-25372-1 (PMC5940663; doi:10.1038/s41598-018-25372-1)
Supplement: Supplementary file 1 — Supplementary Information [file 41598_2018_25372_MOESM1_ESM.pdf]

Supplementary Information

Infants' dietary arsenic exposure during transition to solid food

Antonio J. Signes-Pastor[1,5*], Kathryn L. Cottingham[2,5], Manus Carey[3], Vicki Sayarath[1,5], Thomas Palys[1], Andrew A. Meharg[3], Carol L. Folt[4,5], Margaret R. Karagas[1,5].

[1]Department of Epidemiology, Geisel School of Medicine, Dartmouth College, 1 Medical Center Dr., 7927 Rubin Bldg., Lebanon, NH 03756, USA.

[2]Department of Biological Sciences, Dartmouth College, Hanover, NH 03755, USA.

[3]Institute for Global Food Security, Queen's University Belfast, David Keir Building, Malone Road, Belfast, BT9 5BN, Northern Ireland, UK.

[4]University of North Carolina, Chapel Hill, North Carolina, USA.

[5]Children's Environmental Health and Disease Prevention Research Center at Dartmouth

*Corresponding author's e-mail: antonio.j.signes-pastor@dartmouth.edu

Table S1: Infants' daily intake of food and water (g or mL/day) before (at 4 months of age) and during weaning to solid foods (at 6 months of age).

Food categories and water	4 months of age	6 months of age	6 months of age<br>Percentage of solid food intake
	n [median (min − max)] / (g or mL/day)		*n* [median; mean (min − max)]
Water	4 [172.5 (86.7 − 699.8)]	8 [269.3 (5.0 − 660.3)]	
Formula powder	4 [92.4 (19.4 − 198.5)]	5 [101.3 (18.9 − 330.8)]	
Rice cereal		5 [18.5 (4.7 − 37.9)]	11 [0; 20 (0 − 100)]
Other cereals		6 [40.2 (4.1 − 187.4]	13 [0; 12 (0 − 50)]
Fruits		9 [33.0 (4.7 − 203.2)]	12 [44; 49 (0 − 100)]
Vegetables		7 [42.5 (11.0 − 132.3)]	13 [8; 23 (0 − 87)]
Yougurt		2 [30.7 (4.7 − 56.7)]	13 [0; 3 (0 − 20)]
Other foods*		1 [33.1]	13 [0; 1 (0 − 7)]

*Refers to a participant that reported a meal of turkey with rice without specifying the amount consumed of each product separately.

Table S2: Summary statistics for the observed urinary arsenic species concentrations (µg/L) before (at 4 months of age) and during weaning (at 6 months of age) to solid foods (median (min − max)). These calculations do not consider the paired structure of the data to reflect comparisons of the cohort as a whole at the two time points.

		i-As (µg/L)	MMA (µg/L)	DMA (µg/L)	i-As + DMA+ MMA (µg/L)	AsB (µg/L)
Before weaning (4-month-old) (*n* = 15)		0.109 (0.051 − 0.295)	0.019 (<LOD − 0.188)	0.131 (0.032 − 1.202)	0.265 (0.139 − 1.496)	0.061 (0.016 − 0.241)
During weaning (6-month-old) (*n* = 15)		0.160 (0.049 − 1.453)	0.105 (0.013 − 1.869)	0.759 (0.094 − 9.384)	0.999 (0.170 − 11.950)	0.060 (0.017 − 0.116)
Diff (6 - 4 months of age) (*n* = 15)		0.081 (-0.113 − 1.399)	0.045 (-0.054 − 1.689)	0.430 (-0.709 − 8.476)	0.406 (-0.688 − 10.690)	0.005 (-0.222 − 0.052)
Before weaning (4-month-old)	Breast milk (*n* = 11)	0.101 (0.051 − 0.162)	0.015 (<LOD − 0.021)	0.126 (0.032 − 1.202)	0.241 (0.139 − 1.326)	0.057 (0.016 − 0.139)
	Mixture (*n* = 3)	0.178 (0.068 − 0.295)	0.164 (0.020 − 0.180)	0.661 (0.100 − 0.908)	1.119 (0.188 − 1.266)	0.078 (0.075 − 0.241)
	Formula milk (*n* = 1)	0.151 (0.151 − 0.151)	0.188 (0.188 − 0.188)	1.157 (1.157 − 1.157)	1.496 (1.496 − 1.496)	0.021 (0.021 − 0.021)
During weaning (6-month-old)*	Breast milk (*n* = 10)	0.157 (0.049 − 0.368)	0.053 (0.013 − 0.565)	0.367 (0.094 − 4.055)	0.552 (0.170 − 5.088)	0.058 (0.017 − 0.116)
	Mixture (*n* = 2)	1.076 (0.699 − 1.453)	1.242 (0.614 − 1.869)	7.138 (4.891 − 9.384)	9.456 (6.959 − 11.950)	0.078 (0.061 − 0.096)
	Formula milk (*n* = 3)	0.231 (0.145 − 0.527)	0.203 (0.134 − 0.565)	1.091 (1.005 − 2.179)	1.525 (1.284 − 3.271)	0.019 (0.017 − 0.116)

*At 6 months of age infants’ diet included solid food in addition to breast milk, formula, or a mixture of both breast milk and formula.

Table S3: Estimation of infants' arsenic exposure through household water consumption.

Infant ID	Water arsenic content	4 months of age		6 months of age	
		Daily water intake	Arsenic exposure	Daily water intake	Arsenic exposure
	(µg/L)	(mL)	(µg/day)	(mL)	(µg/day)
P01	0.451			5.0	0.002
P02	10.582				
P03	0.102				
P04	0.044				
P05	0.011				
P06	0.011				
P07	3.801				
P08	6.747				
P09	8.158			21.3	0.174
P10	0.011	699.8	0.008	660.3	0.007
P11	0.011	207.0	0.002	596.0	0.007
P12	0.022/ <1.4*	138.0	0.003/ <0.193	591.3	0.013/ <0.827
P13	0.637			59.0	0.038
P14	8.176			193.7	1.583
P15	0.011	86.7	0.001	345.0	0.004

*Arsenic content reported in bottled water [1].

Figure S1: Spearman's correlation between each food category among weaning infants.

		Formula (g/day)	Rice cereals (g/day)	Other cereals (g/day)	Fruits (g/day)	Vegetables (g/day)	Total solid food (g/day)
Breast feedings [feedings/day]	*n*	15	12	15	13	14	13
	ρ	-0.73	-0.32	-0.70	-0.33	-0.70	-0.71
	p-value	**0.002**	0.30	**0.003**	0.27	**0.004**	**0.006**
Formula (g/day)	*n*		12	15	13	14	13
	ρ		0.42	0.84	0.44	0.80	0.75
	p-value		0.17	**<0.001**	0.13	**<0.001**	**0.003**
Rice cereals (g/day)	*n*			12	10	11	11
	ρ			0.21	0.06	0.41	0.28
	p-value			0.52	0.87	0.21	0.40
Other cereals (g/day)	*n*				13	14	13
	ρ				0.63	0.89	0.85
	p-value				**0.02**	**<0.001**	**<0.001**
Fruits (g/day)	*n*					13	12
	ρ					0.49	0.76
	p-value					0.09	**0.004**
Vegetables (g/day)	*n*						13
	ρ						0.84
	p-value						**<0.001**

rho

-1	0	1

*The amount of food ingested (g/day) refers to the average based on a 3 day food diary.

References:

1. Nestlé Pure Life Nestlé Waters. Bottled Water Quality Report. 1–22 (2012). at <https://www.nestle-watersna.com/asset-library/Documents/PS_ENG.pdf>

3 day food diary template

3-DAY FOOD DIARY – _ MONTHS
Parent Instructions

This diary is designed to record exactly what your baby eats and drinks during the 3 days prior to the urine sample collection. Please record the foods as described below and return the diary and urine sample to us using the enclosed return mailers.

- Each day begins with the first meal after midnight and continues till 11:59 p.m. (just before midnight). Please be sure to write the date at the top of each page.
- For example, if you are collecting the sample on Thursday, start recording after midnight on Monday and continue through the time when you take your baby's urine sample.

Monday	Tuesday<br>Day 1	Wednesday<br>Day 2	Thursday<br>Day 3<br>Doctor Visit

Start: After 12:00am **Stop**: at urine sample

- For each feeding, please record the time at which your baby started eating and then the amounts of **all food and drink** consumed. This includes anything added to foods or drinks, such as water added to powdered formula, milk or formula added to cereals, and supplements added to bottle feeds.
- Record the estimated amount that was **actually consumed**. Do not include liquid that remains in a bottle or food that remains in a bowl after your baby has finished eating.
- For all formulas and ready-made baby foods, be as specific as possible when you record the **brand** (e.g., Similac, Enfamil, Gerber, Target, etc.) and **exact product name** (e.g., Nutrimigen with Enflora LGG, PREMIUM infant, GOODSTART Gentle Formula, etc.).
- If your baby attends daycare or is cared for by someone other than yourself during these 4 days, please provide the caretaker with the diary and ask him/her to complete it during his/her time with your baby.

Sample Diary Entries

BREAST FEEDING: record the number of minutes your baby actually spent feeding.

Time	Food/Drink Item	Amount consumed	Organic? (Yes/No/ Unsure)	Added water (if any)	Water Source, If bottled what brand?
2:00 p.m.	Breastfed, 15 min.				

EXPRESSED BREAST MILK: record the amount that was actually consumed from the bottle.

Time	Food/Drink Item	Amount consumed	Organic? (Yes/No/ Unsure)	Added water (if any)	Water Source, If bottled what brand?
2:00 p.m.	Expressed breastmilk	4 oz.			

READY-TO-USE FORMULA (from a can or bottle): record the name of the product and the amount that was actually consumed.

Time	Food/Drink Item	Amount consumed	Organic? (Yes/No/ Unsure)	Added water (if any)	Water Source, If bottled what brand?
2:00 p.m.	Nestle Good Start Soy Infant Formula w/ Iron, 0-12 months	4 oz.	No		

POWDERED OR CONCENTRATED FORMULAS: record the name of the product and your preparation method. If you use the exact same formula and preparation each time, it is okay to use quotation marks to "carry down" the recipe in the Food/Drink item column, as shown in the example below. However, it is important to write down the source of the water each time if you are using different water sources.

Time	Food/Drink Item	Amount consumed	Organic? (Yes/No/ Unsure)	Added water (if any)	Water Source, If bottled what brand?
2:00 p.m.	Enfamil Premium Newborn powder formula: 2 scoops	2 scoops	No	4 oz.	Restaurant
4:00 p.m.	"	"	"	"	tap
9:00 p.m.	"	"	"	"	tap

CEREALS/WHOLE GRAINS PREPARED WITH WATER, MILK OR FORMULA (hot infant cereals [rice, wheat, oatmeal] or pureed rice/grains)**:** record your recipe in the recipes section at the back of the diary (including information about Added water, if any), then indicate the recipe number to which you're referring and the amount consumed in the diary.

Time	Food/Drink Item	Amount consumed	Organic? (Yes/No/ Unsure)	Added water (if any)	Water Source, If bottled what brand?
2:00 p.m.	Solid Food 2	3 Tbsp.	No		

OTHER SOLID FOODS: If store-bought, record the product information (brand and product name). If homemade, please record your recipe in the recipes section at the back of the diary and then indicate the recipe to which you are referring and the amount consumed in the main diary. If adding water when serving the food, please include the amount of water added to the food in the "Added water" column and also record its source. If your baby is eating food for which you do not have the exact recipe (for example, you are at a friend's or relative's, or out at a restaurant), note that you don't have the exact recipe and try to write down what you think the major ingredients are, as best you can.

Time	Food/Drink Item	Amount consumed	Organic? (Yes/No/ Unsure)	Added water (if any)	Water Source, If bottled what brand?
2:00 p.m.	Earth's Best Organic 1st apples	1 Tbsp.	Yes	1 Tbsp.	Tap
2:00 p.m.	Solid Food 1	2 Tbsp.	No		

VITAMINS/SUPPLEMENTS: record the product information and the amount added in drops, vials, tablets, etc. Record the supplement in the same food entry for which the supplement was added.

Time	Food/Drink Item	Amount consumed	Organic? (Yes/No/ Unsure)	Added water (if any)	Water Source, If bottled what brand?
2:00 p.m.	Expressed breast milk<br><br>Enfamil human milk fortifier acidified liquid	3 oz.<br><br>1 vial		No	--

CONCENTRATED JUICES: record the name of the product and your preparation method. If you feed your baby this same juice again, indicate as shown below. If adding extra water to the prepared juice, please list the amount in the “Added water” column.

Time	Food/Drink Item	Amount consumed	Organic? (Yes/No/ Unsure)	Added water (if any)	Water Source, If bottled what brand?
2:00 p.m.	Welches White Grape Peach 100% Juice, frozen: 11.5 oz can + 3 cans water	3 oz.	No		Tap
4:00 p.m.	SAME white grape juice	2 oz.		1 oz.	"

Once complete, place the diary into the enclosed plastic bag and bring it to your pediatrician’s office along with the urine and diaper samples for your 4-month visit. A coordinator will meet you there to pick it up.

Thank you for your participation in the New Hampshire Birth Cohort Study! ☺

EXAMPLE Solid Food Recipes:

Solid Food 1: Sweet potatoes & Pears

Ingredients		
**Item Description**	**Quantity**	**Organic? (Yes/No/ Unsure)**
Medium peeled pear	2	Y
Large peeled sweet potato	1	N
_	_	_
_	_	_
_	_	_

Did you add water? If yes, what was the source? Home tap **How much did you add?** 2 cups
Total prepared volume: 2.5 cups
Preparation method:
Pears and sweet potato were boiled in water and then mashed together with remaining water.

Solid Food 2: Pureed brown rice

Ingredients		
**Item Description**	**Quantity**	**Organic? (Yes/No/ Unsure)**
Lundburg organic short grain brown rice	½ C.	yes
_	_	_
_	_	_
_	_	_

Did you add water? If yes, what was the source? Tap **How much did you add?** 1 c. .
Total prepared volume: 1 1/4 c. .
Preparation method:
Rice was boiled in water for 45 minutes till water was absorbed, then pureed in a food processor.

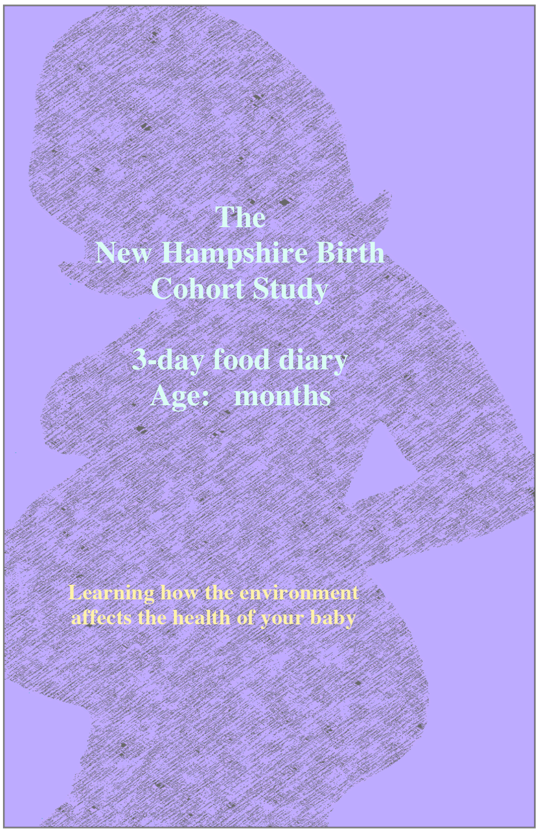

Thank you for your participation in the New Hampshire Birth Cohort Study! ☺

Day 1: ___ / ___ / ___

Month Day Year

Day begins at 12:00 a.m. (midnight) and ends at 11:59 p.m. the next day.

Time	Food/Drink Item	Amount consumed	Organic? (Yes/No/Unsure)	Amount of added water (if any)	Water source, if bottled what brand?

Day 1 continued…

Day begins at 12:00 a.m. (midnight) and ends at 11:59 p.m. the next day.

Time	Food/Drink Item	Amount consumed	Organic? (Yes/No/Unsure)	Amount of added water (if any)	Water source, if bottled what brand?

Day 2: ___ / ___ / ___

Month Day Year

Day begins at 12:00 a.m. (midnight) and ends at 11:59 p.m. the next day.

Time	Food/Drink Item	Amount consumed	Organic? (Yes/No/Unsure)	Amount of added water (if any)	Water source, if bottled what brand?

Day 2 continued…

Day begins at 12:00 a.m. (midnight) and ends at 11:59 p.m. the next day.

Time	Food/Drink Item	Amount consumed	Organic? (Yes/No/Unsure)	Amount of added water (if any)	Water source, if bottled what brand?

Day 3: ___ / ___ / ___

Month Day Year

Day begins at 12:00 a.m. (midnight) and ends at your doctor's appointment.

Time	Food/Drink Item	Amount consumed	Organic? (Yes/No/Unsure)	Amount of added water (if any)	Water source, if bottled what brand?

Day 3 continued…

Day begins at 12:00 a.m. (midnight) and ends at your doctor’s appointment.

Time	Food/Drink Item	Amount consumed	Organic? (Yes/No/Unsure)	Amount of added water (if any)	Water source, if bottled what brand?

Solid Food Recipes:

Solid Food 1: ____

Ingredients		
Item Description	Quantity	Organic? (Yes/No/ Unsure)

Did you add water? If yes, what was the source? ______________ **How much did you add?** _____________

Total prepared volume: ____________

Preparation method:

Solid Food 2: ____

Ingredients		
Item Description	Quantity	Organic? (Yes/No/ Unsure)

Did you add water? If yes, what was the source? ______________ **How much did you add?** _____________

Total prepared volume: ____________

Preparation method:

Solid Food Recipes:

Solid Food 3: ____

Ingredients		
**Item Description**	**Quantity**	**Organic? (Yes/No/ Unsure)**

Did you add water? If yes, what was the source? _____________ **How much did you add?** ____________
Total prepared volume: ___________
Preparation method:

Solid Food 4: ____

Ingredients		
**Item Description**	**Quantity**	**Organic? (Yes/No/ Unsure)**

Did you add water? If yes, what was the source? _____________ **How much did you add?** ____________
Total prepared volume: ___________
Preparation method:

Solid Food Recipes:

Solid Food 5: ____

Ingredients		
**Item Description**	**Quantity**	**Organic? (Yes/No/ Unsure)**

Did you add water? If yes, what was the source? _____________ **How much did you add?** ____________
Total prepared volume: ___________
Preparation method:

Solid Food 6: ____

Ingredients		
**Item Description**	**Quantity**	**Organic? (Yes/No/ Unsure)**

Did you add water? If yes, what was the source? _____________ **How much did you add?** ____________
Total prepared volume: ___________
Preparation method:

Solid Food 7: ____

Ingredients		
Item Description	Quantity	Organic? (Yes/No/ Unsure)

Did you add water? If yes, what was the source? ____________ How much did you add? ___________

Total prepared volume: __________

Preparation method:

Solid Food 8: ____

Ingredients		
Item Description	Quantity	Organic? (Yes/No/ Unsure)

Did you add water? If yes, what was the source? ____________ How much did you add? ___________

Total prepared volume: __________

Preparation method:

Solid Food 9: ____

Ingredients		
Item Description	Quantity	Organic? (Yes/No/ Unsure)

Did you add water? If yes, what was the source? _____________ How much did you add? ____________
Total prepared volume: ___________
Preparation method:

Solid Food 10: ____

Ingredients		
Item Description	Quantity	Organic? (Yes/No/ Unsure)

Did you add water? If yes, what was the source? _____________ How much did you add? ____________
Total prepared volume: ___________
Preparation method:

Solid Food Recipes:

Solid Food 11: ____

Ingredients		
Item Description	Quantity	Organic? (Yes/No/ Unsure)

Did you add water? If yes, what was the source? ______________ **How much did you add?** _____________
Total prepared volume: ____________
Preparation method:

Solid Food 12: ____

Ingredients		
Item Description	Quantity	Organic? (Yes/No/ Unsure)

Did you add water? If yes, what was the source? ______________ **How much did you add?** _____________
Total prepared volume: ____________
Preparation method:

Solid Food Recipes:

Solid Food 13: ____

Ingredients		
**Item Description**	**Quantity**	**Organic? (Yes/No/ Unsure)**
_	_	_
_	_	_
_	_	_

Did you add water? If yes, what was the source? ______________ **How much did you add?** _____________
Total prepared volume: ____________
Preparation method:

Solid Food 14: ____

Ingredients		
**Item Description**	**Quantity**	**Organic? (Yes/No/ Unsure)**
_	_	_
_	_	_
_	_	_

Did you add water? If yes, what was the source? ______________ **How much did you add?** _____________
Total prepared volume: ____________
Preparation method:
